# Assessing temporal correlation in environmental risk factors to design efficient area-specific COVID-19 regulations: Delhi based case study

**DOI:** 10.1038/s41598-022-16781-4

**Published:** 2022-07-28

**Authors:** Vishal Chaudhary, Pradeep Bhadola, Ajeet Kaushik, Mohammad Khalid, Hidemitsu Furukawa, Ajit Khosla

**Affiliations:** 1 Research Cell and Department of Physics, Bhagini Nivedita College, University of Delhi, New Delhi, 110043 India; 2 Centre for Theoretical Physics and Natural Philosophy, Nakhonsawan Studiorum for Advanced Studies, Mahidol University, Nakhonsawan, 60130 Thailand; 3 NanoBioTech Laboratory, Health System Engineering, Department of Environmental Engineering, Florida Polytechnic University, Lakeland, FL 33805 USA; 4 School of Engineering, University of Petroleum and Energy Studies (UPES) , Dehradun, Uttarakhand India; 5 Graphene and Advanced 2D Materials Research Group (GAMRG), School of Engineering and Technology, Sunway University, No. 5, Jalan University, Bandar Sunway, 47500 Petaling Jaya, Selangor Malaysia; 6 Sunway Materials Smart Science & Engineering (SMS2E) Research Cluster, Sunway University, No. 5, Jalan Universiti, Bandar Sunway, 47500 Petaling Jaya, Selangor Malaysia; 7 Department of Mechanical Systems Engineering, Graduate School of Science and Engineering, Yamagata University, Yonezawa, Yamagata 992-8510 Japan; 8 School of Advanced Materials and Nanotechnology, Xidian University, Xi’an, 710126 People’s Republic of China

**Keywords:** Environmental sciences, Diseases, Mathematics and computing

## Abstract

Amid ongoing devastation due to Serve-Acute-Respiratory-Coronavirus2 (SARS-CoV-2), the global spatial and temporal variation in the pandemic spread has strongly anticipated the requirement of designing area-specific preventive strategies based on geographic and meteorological state-of-affairs. Epidemiological and regression models have strongly projected particulate matter (PM) as leading environmental-risk factor for the COVID-19 outbreak. Understanding the role of secondary environmental-factors like ammonia (NH_3_) and relative humidity (RH), latency of missing data structuring, monotonous correlation remains obstacles to scheme conclusive outcomes. We mapped hotspots of airborne PM_2.5_, PM_10_, NH_3_, and RH concentrations, and COVID-19 cases and mortalities for January, 2021-July,2021 from combined data of 17 ground-monitoring stations across Delhi. Spearmen and Pearson coefficient correlation show strong association (p-value < 0.001) of COVID-19 cases and mortalities with PM_2.5_ (r > 0.60) and PM_10_ (r > 0.40), respectively. Interestingly, the COVID-19 spread shows significant dependence on RH (r > 0.5) and NH_3_ (r = 0.4), anticipating their potential role in SARS-CoV-2 outbreak. We found systematic lockdown as a successful measure in combatting SARS-CoV-2 outbreak. These outcomes strongly demonstrate regional and temporal differences in COVID-19 severity with environmental-risk factors. The study lays the groundwork for designing and implementing regulatory strategies, and proper urban and transportation planning based on area-specific environmental conditions to control future infectious public health emergencies.

## Introduction

The suddenness and global scope of the Serve Acute Respiratory Coronavirus2 (SARS-CoV-2) outbreak have stressed the designing pandemic retarding modalities to control the devastation^1,2^. These contagion restricting policies includes unprecedented strict lockdown, social distancing, wearing face-masks, massive vaccination, and disinfecting strategies^3–7^. Most research is dedicated to modelling advanced point-of-care diagnostic practices and treatment therapeutics to control pandemic-instigated morbidities and mortalities^3,8–13^. However, the success of employed strategies has been found to vary spatially and influenced by diverse regional environmental factors^14–17^. Moreover, some regions worldwide have been identified as SARS-CoV-2 hotspots and are severely affected by contagion ^18,19^. The mutable nature of SARS-CoV-2 due to its capricious spread, different transmission routes, and its interaction with environmental elements, is challenging prevailing preventive strategies, including massive vaccination. Hence, a critical global public health objective is to identify key tunable regional factors contributing to variable coronavirus disease (COVID-19) severity.

Numerous epidemiological and regression studies have anticipated different regional variables, including geographical, seasonal, demographic, and environmental factors determining the COVID-19 impact^17,20–23^. Air contamination has been identified as the crucial factor governing regional SARS-CoV-2 spread and severity^24–28^. Air contamination is a complex mixture of particulate and gaseous constituents like particulate matter (PM), nitrous oxides (NO_2_), ammonia (NH_3_), sulfur dioxide (SO_2_), and carbon oxides (CO_x_), ozone (O_3_) that varies temporally and spatially. The air quality index (AQI) depends upon various geographical, economic, and demographic conditions^24–28^. Moreover, the urban pollution due to anthropogenic source pollutants or combustion of traffic-related products induces airway hyper-responsiveness and inflammation resulting in the severity of cardiovascular and respiratory diseases^29–32^.

Doremalen et al*.*^33,34^ have reported that COVID-19 is also a respiratory disease, and SARS-CoV-2 remains compelling and infectious in aerosols for a prolonged time. Several In vitro and In vivo studies have demonstrated that exposure to air contaminants decreases immune response facilitating viral replication and penetration^25,27^. Moreover, the complex multi-interactions amongst virus and air contaminants through covalent and electrostatic interactions promote SARS-CoV-2 persistence in the atmosphere and reduce vitamin-D synthesis^35^. Atmospheric aerosols resulting from these interactions induce indirect hazards in the human body, alter the immune response, and are responsible for pro-inflammatory and oxidative mechanisms in the lungs.

Numerous outdoor air contaminants, including ammonia, particulate matter (PM), sulfur dioxide, nitrogen oxides, and carbon oxides, have been argued to possess different roles in COVID-19 transmissibility^27,28,32,36^. Amongst all, PM is anticipated to be most severe factor, which constitute fine particles possessing a primary (car exhaust, construction, and road traffic) and a secondary origin (including ammonia, oxides of nitrogen, and sulfur, which transform into particles through photochemical reactions in the atmosphere)^29,37–41^. Numerous scientific findings reviewed by the US Environmental Protection Agency (EPA)^42–45^ have related fine PM particles (PM_2.5_; PM with diameter ≤ 2.5 µm) to diversified adverse health issues, including mortality. The virus's droplets are argued to bind with PM, promoting the diffusion of droplets with the SARS-CoV-2 in aerosols^36^. The virus particles bound to minute PM like PM_2.5_ are probable to penetrate deeper in the alveolar and tracheobronchial regions of the susceptible host individual^27,36^. Additionally, PM plays an indirect role in COVID-19 spread and impact by weakening the immune system and increasing individual vulnerability towards this disease^46,47^. It is argued to facilitate SARS-CoV-2 binding with susceptible cells by stimulating overexpression of ACE-2 receptors^48–51^. The impaired immune ability and chronic inflammation of individuals living in hot -spots of PM have already been documented^38,47,52–54^. Additionally, the secondary air contaminants like ammonia, NO_x_ and tropospheric O_3_ contribute to PM formation and directly relate to COVID-19 spread and impact^54^. Moreover, the nature and concentration of particular air contaminants, meteorological conditions like rainfall, humidity, temperature, and demographic factors govern their interactions, viral persistence, transmission, and severity.^55,56^.

Various regression analyses have suggested a correlation between air contaminants and COVID-19 mortalities and concluded that the individuals living in polluted areas are more susceptible to COVID-19^57^. Moreover, the megacities (like Delhi, Mumbai, London, New York) were severely affected by SARS-CoV-2 during COVID-19 waves^18^. Furthermore, during the pandemic, India has documented a rise in several emerging and re-emerging infectious diseases, which has created more burdens on the healthcare system and vaccination drive^58–62^. The pollution level in Delhi is reported to decrease by 15% from 2019 to 2020 due to government policies, public awareness, strict lockdown, and alternative pollution combating solutions^60^. Nevertheless, according to the World Air Quality report 2020, released by IQAir, Delhi is the most polluted capital globally, with an average annual PM_2.5_ concentration of 84.1 µg per cubic meter^58^.

Various reports in the literature have explored the correlation between COVID-19 severity and AQI during the first COVID-19 wave in different megacities^25,27,63–65^. However, to the best of our knowledge, no report is dedicated explicitly to evaluating PM and ammonia's impact on COVID-19 spread and severity during the second COVID-19 wave in Delhi. Moreover, various noticeable methodological difficulties have been pointed out in different studies to establish this correlation^66–70^. Ideally, the study should be comprehended to emphasise the variability of specific contaminants. The other variables can be mutually highly correlated, which presents an obstacle for any statistical interference analysis^70–73^. To address, it we employed two correlation strategies to analyze the correlation between PM and COVID-19 severity in terms of RH and ammonia during the second COVID-19 wave in Delhi. We aim to provide fundamental reasoning to design regional-based strategies depending upon environmental variables including PM, RH, and NH_3_ to prevent and control COVID-19 severity and future epidemic devastations.

## Results

### Analysis to map hotspots of environmental risk factors

**Figure 1:**
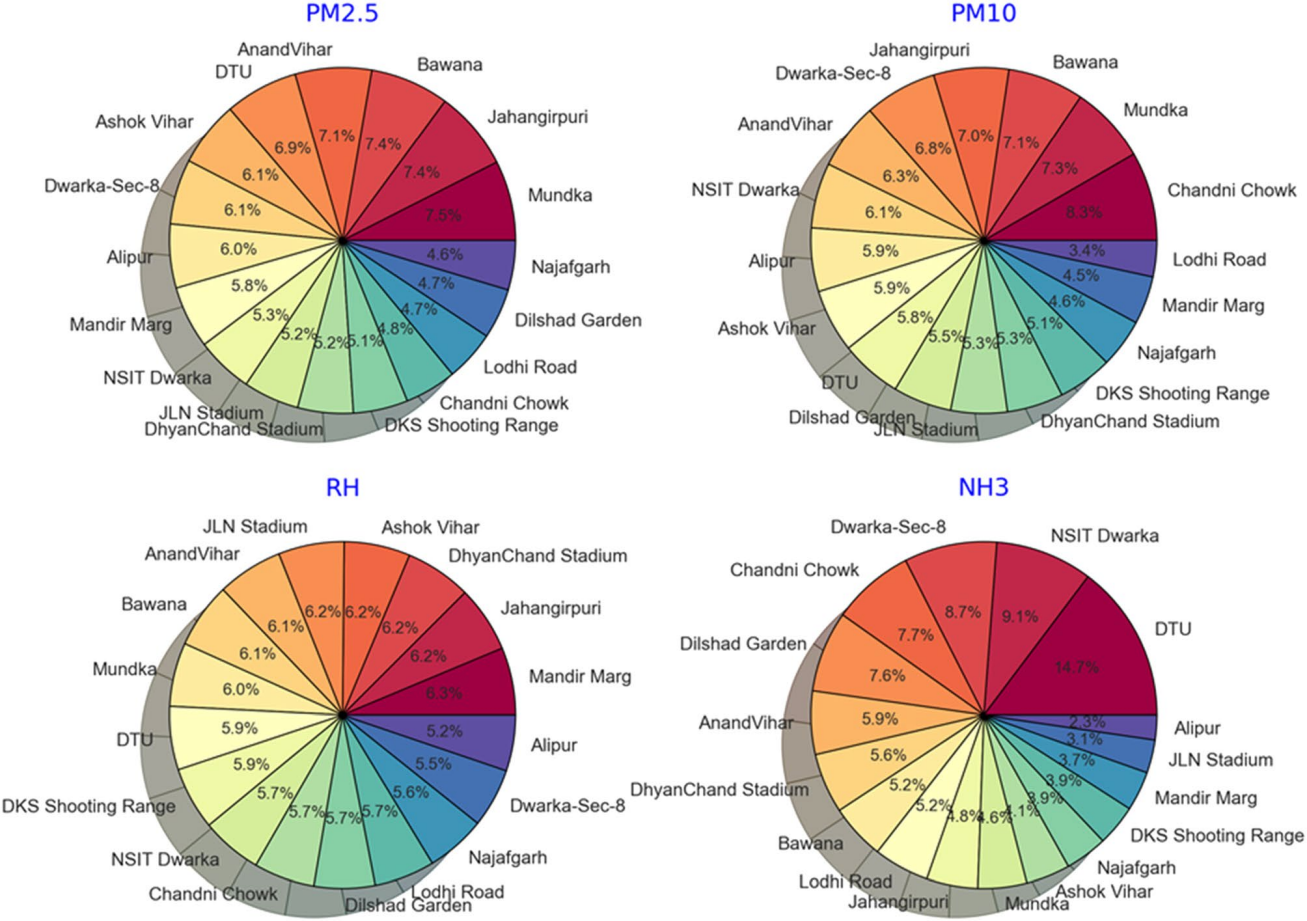
Pie chart showing the regional distribution of percentage contribution of variables observed from all sub-station locations to the overall AQI of Delhi to locate particular hotspots.

The present trend in the Delhi region straightaway revealed the potential differences at the regional or sub-station level for reported AQI values. The contribution of pollutant concentration in Delhi's atmosphere from 17 monitoring sub-stations varies regionally, as shown in Fig. 1.

Amongst all sub-stations in Delhi, Mundka, Jahangirpuri, and Bawana regions show the highest average (From Jan to July 2021) contribution for PM_10_ and PM_2.5_, except for Chandni Chowk, which has the highest PM_10_ is one of the lowest contributors among PM_2.5_. The average contribution from the Mundka, Jahangirpuri, and Bawana regions towards atmospheric PM_2.5_ is estimated to be around 129 µg/cm^3^. All the three higher PM_2.5_ contributors (Mundka, Jahangirpuri, and Bawana) have been identified as industrial areas of Delhi^74,75^. These areas are surrounded by green areas/agricultural regions of Delhi, which contribute to the emission of fine organic particles into the environment. However, the exceptional presence of the Chandni Chowk region, identified as the highest PM_10_ contributor, can be attributed to the recent construction performed in the region for its ongoing beautification and redevelopment.

It is documented that long-term exposure to PM_2.5_ increases the morbidity and mortality rate due to COVID-19^25,49,50^. The pathogenicity of PM is strongly determined by its chemical compositions of particles like trace elements. The trace elements such as arsenic (As), cadmium (Cd), chromium (Cr), lead (Pb), and mercury (Hg) represent a minute fraction of net PM mass^38,51,52^. However, the concentrations are adequate to injure human health, like cardiopulmonary or lung injuries, neurodegenerative diseases, and low birth weight. Recently, Tobler et al.^76^ revealed PM's chemical composition and source attribution in the Delhi-National capital region (NCR). The coarse fraction (PM_10_) was sourced by organic matter (OM), sulfur- nitrate-ammonium (SNA) ions, and crustal materials. However, the fine fraction (PM_2.5_) was dominated by OM, SNA ions, and elemental carbon. Further, Hama et al*.*^77^ revealed that the construction and paved road dust are major contributing factors to PM and are dominated by the most abundant elements, including K, Si, Fe, Al, and Ca. Hence, the analyzed chemical compositions of PM in various reports suggest mixed sources of PM, including vehicular emission, construction, biomass burning, and paved road dust. Hence, the prevalence of these PM hotspots is attributed to contributions from vehicular emission construction work and OM suspended aerosols from agricultural practices^78^.

The observed regional variation of ammonia in the Delhi region is different from that for PM. Among all Delhi sub-stations, DTU, NSIT Dwarka, and Dwarka regions are the topmost contributors to airborne ammonia. These top three regions contribute an average of one-third (over 32%) of airborne ammonia over Delhi. DTU region has been observed to contribute 14% of total airborne ammonia in overall Delhi. The primary source of airborne ammonia is the agricultural sector, including livestock, agricultural fields, and biomass degradation^79,80^. DTU and Dwarka region is surrounded by agricultural fields and is greener than the rest of the Delhi regions. Hence, their observed role as the highest contributor to airborne ammonia is justified.

In the present study, the regional variation in relative humidity (RH) has also been analyzed. The RH of all regions is nearly the same, with not much variance. It can be attributed to similar weather conditions throughout the Delhi region due to its comparatively smaller area. It reveals the interannual variability of RH depending enormously on the reference year implying a dominant meteorological influence in Delhi.

Hence, the spatial variation of observed variables (PM, NH_3_, and RH) in Delhi has been observed, and the appropriate reasoning has been illustrated. It signifies that the regions with higher industrial and agricultural sectors in Delhi contribute to more significant variable concentrations and are identified as hotspots for respective variable emissions. It also anticipates the variation of impacts of these pollutants on the atmosphere and living beings in Delhi.

### Statistical analysis during the second COVID-19 impact wave

The statistics of variables (PMs, RH, COVID-19 mortality, and cases) as average over the period (January 2021 to July 2021) have been estimated. The average of variables over the considered period has been considered to analyze their correlation for the whole Delhi region. The mean values of PM2.5 and PM10 for the considered period are moderately and very polluted, respectively, as per guidelines of the National AQI (Table 1). The maxima of both PMs lie in the abysmal AQI range, which raises severe public health and environmental concerns. However, the average value of ammonia over the considered period is in the acceptable range, causing no severe concern. The simultaneous existence of low ammonia concentration and higher PM concentration can be attributed to the photochemical formation of PM in the atmosphere at the cost of ammonia^41^. This COVID-19 period in Delhi has been considered as its second significant impact wave, costing approximately 74 lives per day in the aforesaid period. The average confirmed cases are extensive at approximately 4,153 per day, which indicates the rapid spread of COVID-19 in the period described above in Delhi. Hence, the average value of COVID-19 cases and mortalities show the severe impact of COVID-19 on Delhi in the considered period and justify the choice of the aforesaid period for analysis.

**Table 1:**
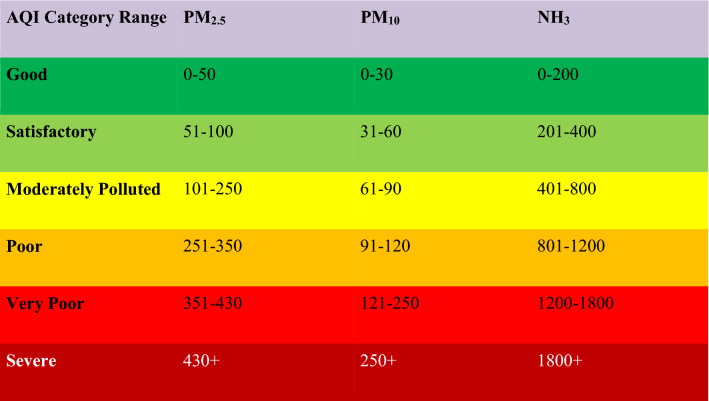
Category range of AQI for considered pollutants as per National AQI, CPCB (2014) illustrating AQI categories advisable for human health in India.

**Figure 2:**
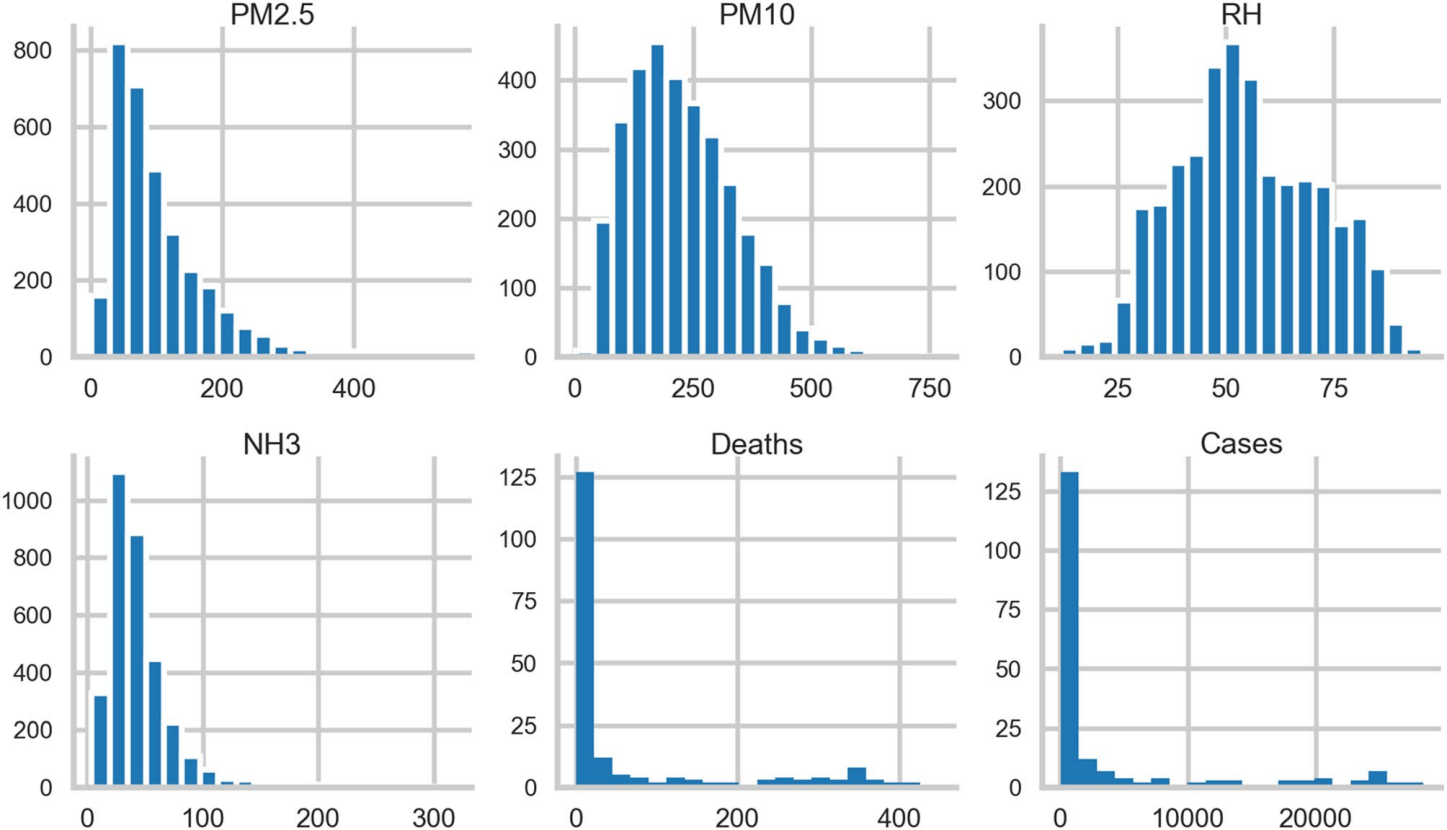
Distributions of the variables for the entire period with variable value at x-axis and frequency of that value over all locations at the y-axis.

Further, the graphical distribution of all the variables for the entire considered period has been represented in Fig. 2.

**Figure 3:**
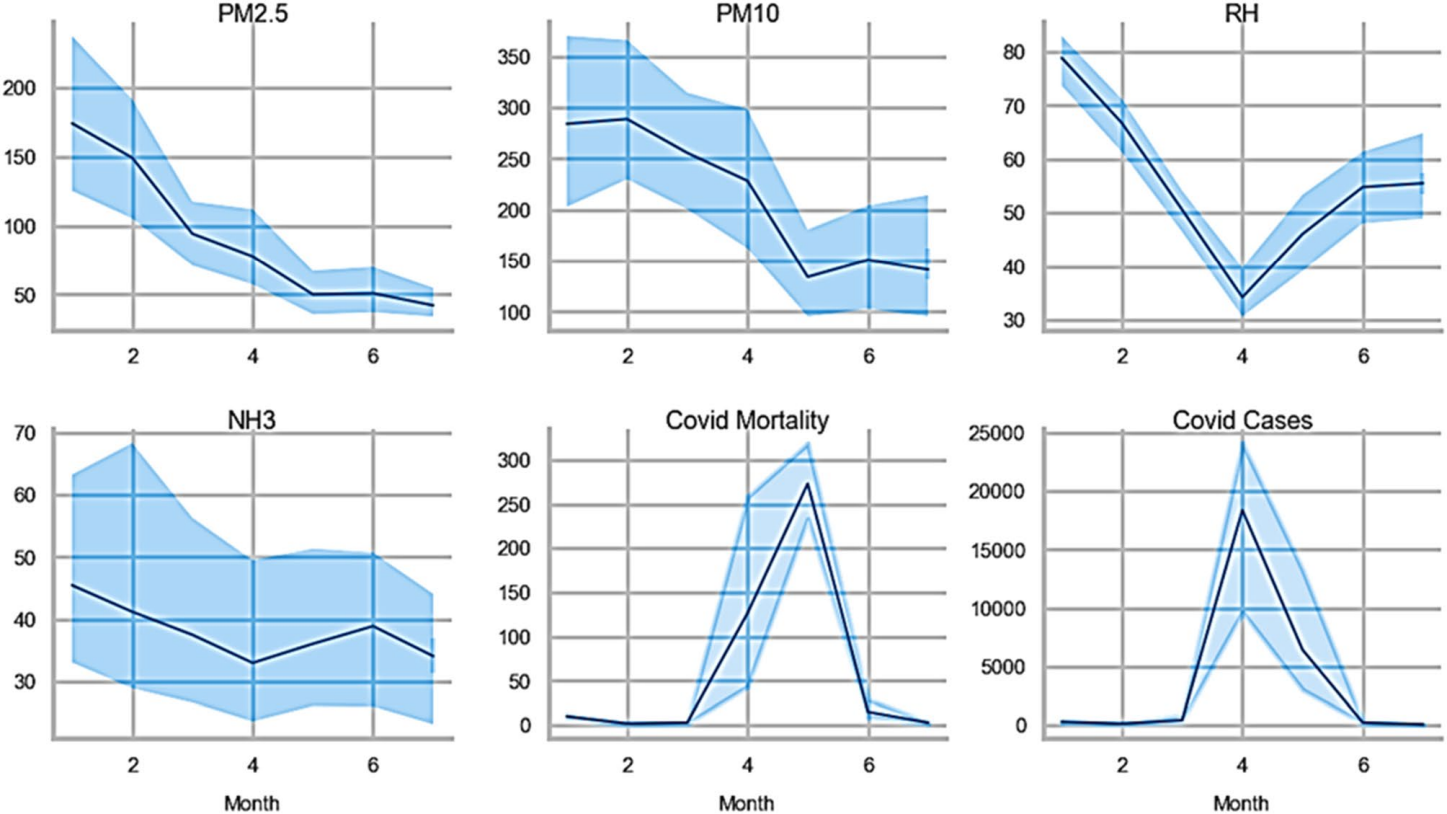
Month-wise distribution of variables including environmental risk factors and COVID-19 severity through-line plot.

**Figure 4:**
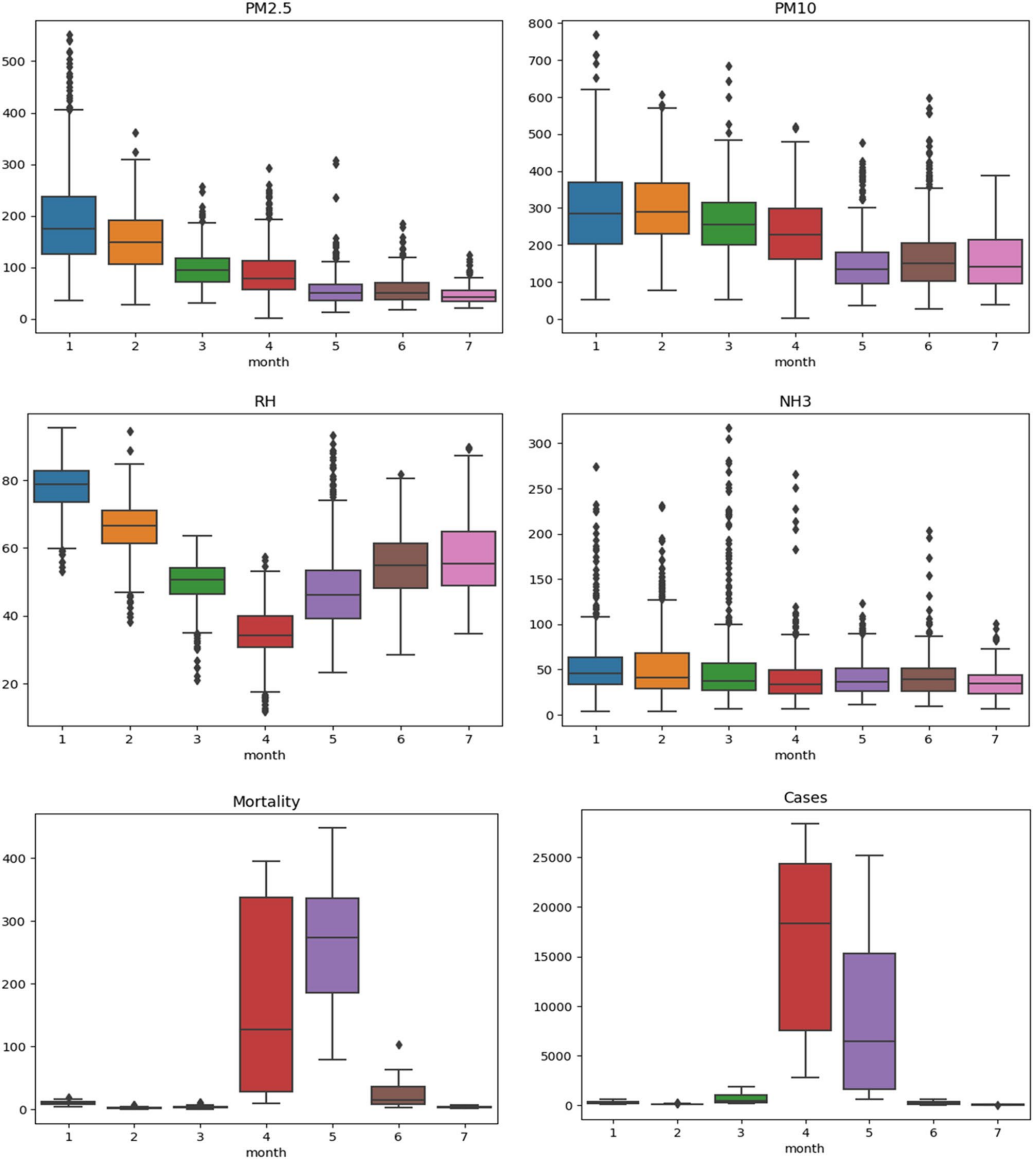
Box plot for month-wise distribution of variables including environmental risk factors and COVID-19 severity.

Further, the data has been analyzed month-wise to observe the exact nature and period of the second COVID-19 impact wave in Delhi. The data for pollutants has also been examined month-wise to perceive the concentration of pollutants during the second COVID-19 impact wave. The month-wise distribution of variables is illustrated as Line Plot (Fig. 3) and Box plot (Fig. 4) for better understanding.

The mean level of each pollutant within the considered aforesaid period decreased from January 2021 to July 2021. It is attributed to the dependence of PM on meteorological conditions^74,76^. It is evident from the literature that the winter season is favourable for the formation of PM^78,81^.

Moreover, paddy stubble burning during the winter in Delhi-NCR regions and adjacent states raises the PM concentration^82^. Hence, the concentration of PM in the Delhi atmosphere is comparatively more in January 2021 than in July 2021. A similar pattern of decrease in RH with time was observed for January 2021 to March 2021, which is the later spring season in Delhi. However, the RH value was found to again increase from April 2021 to July 2021, which is attributed to the onset of the summer season in Delhi.

It has already been reported that the air pollutant concentration varies smoothly with changes in RH^83^. Hence, their correlation analysis is of significant importance. The concentration of ammonia in Delhi's environment was found to follow the same pattern as RH. It is found to decrease with time from January 2021 to March 2021 and then increase from March 2021 onwards. During the aforesaid period, the decrease in pollutant concentrations is attributed to preventive measures like strict lockdown imposed by the government.

Moreover, it has been observed that there is a sudden increase in COVID-19 cases and mortalities during April 2021. This period is identified as the second significant COVID-19 impact wave in Delhi. However, the COVID-19 impact showed a downward trend in the count of COVID-19 cases and mortalities during the lockdown phase (19th April 2021 to 31st May 2021). It shows that the lockdown was proven efficient in controlling the COVID-19 impact in Delhi during the second wave.

### Analysis of pairwise relation amongst the different variables

**Figure 5:**
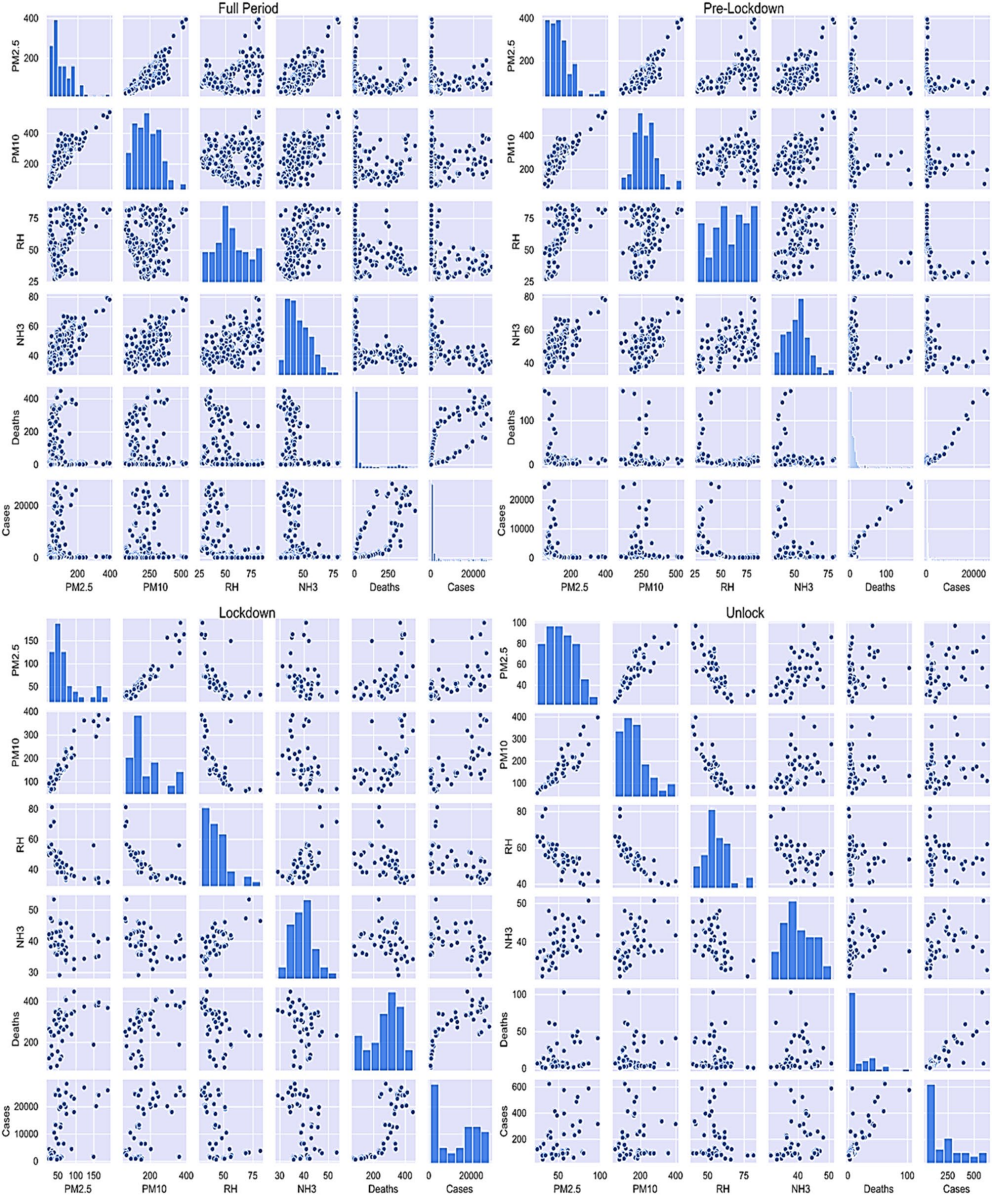
Pairwise relationship of different variables including environmental risk factors and COVID-19 severity for entire period, pre-lockdown period, lockdown period, and unlock period.

The entire considered period has been divided into three-time frames, including pre-lockdown (1st January 2021 to 18th April 2021), lockdown (19th April 2021 to 31st May 2021), and unlock (1st June 2021 to 31st April) periods based on lockdown imposed by the government in Delhi. The pairwise relation has been evaluated for all the periods described above for different variables, as shown in Fig. 5. The diagonal subplots show the distribution of each variable, whereas the off-diagonal subplots show the pairwise relationship between variables. A linear dependence between PM10 and PM2.5 has been obtained for the considered period. It can be attributed to the standard climatic requirements and the chemical composition of PMs. Similarly, a linear relationship between COVID-19 cases and mortalities has been observed during the pre-lockdown and unlock period. However, there is moderate relative dependence between the two during the lockdown period. The restriction imposed on residents during the lockdown period significantly contributed to controlling the COVID-19 spread.

Moreover, the imposed restrictions during the lockdown, including restrictions on movement and restricting construction and industrial activities, have improved the air quality of the Delhi atmosphere^60,78^. Hence, the relationship between the COVID-19 and mortalities slightly diverges from linear dependence. However, the observed dependence of COVID-19 impact on RH and pollutants' concentration was not linear. Hence, it indicates that Pearson correlation coefficient analysis may result in discrepancies, which can be considered by employing Spearman analysis.

### Analysis of normalized variables with time

All the variables are normalized prior to comparative analysis. In a typical normalization process, all the variables are rescaled such that they have zero mean and unit variance such that:

$${\text{New variable:}}{\text{ Yi}}\, = \,\frac{{x_{i} - \mu_{x} }}{\sigma \left( x \right)}$$

where x_i_ is the old variable, µ is the mean, and σ(x) is the variance.

**Figure 6:**
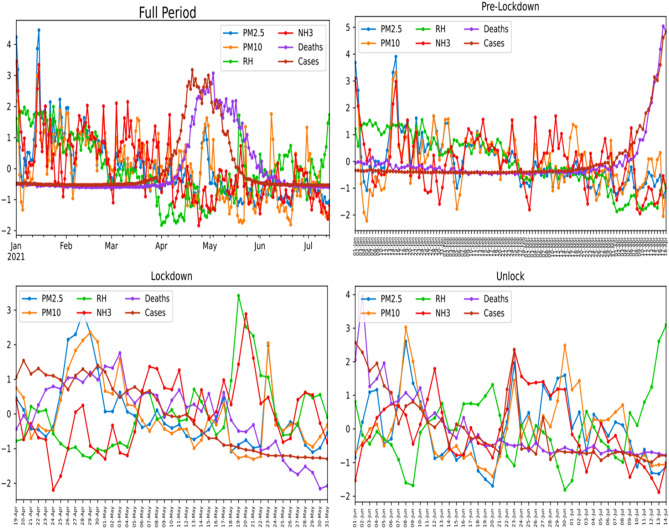
Variation of normalized variables with time for full period, pre-lockdown period, lockdown period, and unlock period.

The evaluated variation of these normalized variables with time for the different considered periods has been illustrated in Fig. 6. It is done to check and compare the variables during different periods.

It is evident from Fig. 6 that the COVID-19 impact and transmission are higher in the region where RH is low. Relative humidity (RH) and atmospheric temperature play a crucial role in the progression and persistence of the virus in the atmosphere. RH affects virus transmission in three significant ways^55,56^. It includes the human immune system's enhanced capability in higher RH, the SARS-CoV-2 decays faster when RH is around 60%, and drier air (low RH) results in further transmission of the virus along with deeper penetration into the human lungs. Thus, this reasoning further validates our observation of the rise in COVID-19 impact in terms of cases and mortalities with RH.

During the pre-lockdown period, the pollutant variation shows significant variance with large fluctuations. The pre-lockdown peaks for PMs and NH_3_ were observed on 14th January 2021. The random fluctuations during this period can be ascribed to various pollutant contributing factors, including biomass burning, use of agricultural fertilisers, construction and roadside-based dust, industrial emission, and vehicular emission. The more significant number of contributing factors during the pre-lockdown interval has resulted in more substantial fluctuations in recorded pollutant concentrations.

A sudden increase in COVID-19 impact cases and mortalities during April 2021 is evident in Fig. 6, which is considered the commencement of the second wave in Delhi. It has led to a strict lockdown in Delhi as a precautionary measure to combat the COVID-19 impact. PMs showed the highest concentration values during the lockdown period on 28–29 April 2021, whereas RH and NH_3_ showed elevated peaks on 19–20 May 2021. The COVID-19 impact (cases and mortalities) was also highest around similar dates (26–30 April 2021), which indicates a correlation between PM level and COVID-19 impact. It has also been observed that the COVID-19 cases and mortalities were higher during the initial lockdown phase and started to decline towards the end of the lockdown phase. COVID-19 is a highly infectious and communicable respiratory disease^3^. It is observed to control significantly by imposing lockdown in Delhi by Delhi-NCT and the Central government of India^60^. The restrictions on movement, industrial, construction, and commercial activities have helped improve Delhi's AQI. It restricts the primary and secondary transmission route of the SARS-CoV-2 virus through respiration, aerosols, and infected surfaces. It also results in combating the immediate impact of pollutant inhalation/absorption in the human body, such as weakening the immune system and affecting the respiratory system. A similar trend in variation of PM with time in the lockdown phase has been observed, which suggests the existence of a strong correlation between PM with COVID-19 impact.

However, even after imposing the lockdown on April 19, the COVID-19 cases as well as mortalities still shows a rise and peaks at around 1st May 2022. The increase in the cases, as well as mortalities even after lockdown, can be attributed to the incubation period for COVID-19. After 1st May we see a clear downward trend in mortalities Fig. 6, which is clearly the effect of lockdown.

When a significant decrease in deaths and cases during the lockdown period is observed, Delhi and Central Government implement a phase-wise unlock period. During the unlock Phase, multiple peaks are observed in the pollutant levels (PMs, NH3). The COVID-19 cases and deaths continue to decline during the unlock Phase suggesting the proper implementation of government policies regarding step-wise unlock phases to combat COVID-19 impact in terms of transmission and mortalities.

### Correlation analysis

**Figure 7:**
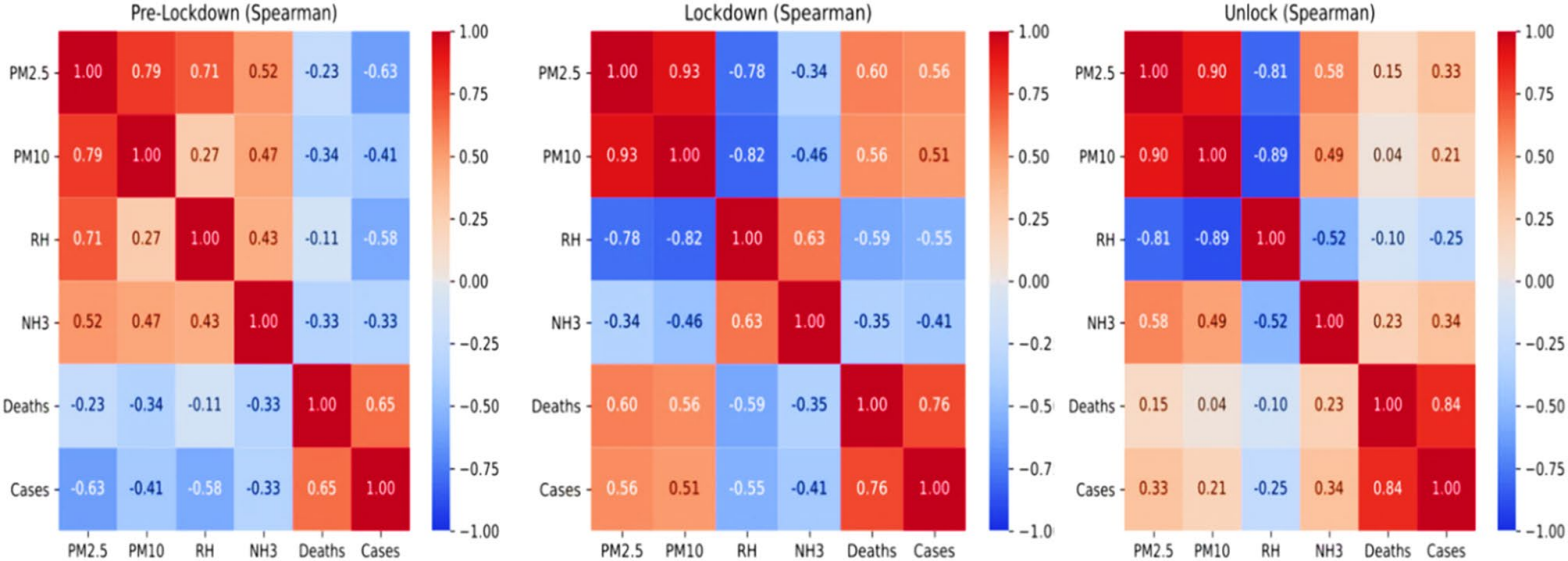
Spearman correlation plots amongst different variables for pre-lockdown phase, lockdown phase, and unblock phase.

The variation from linear dependence relationships amongst the different variables for the considered period has suggested proper strategies for correlation analysis. It is taken into account by choosing the Spearmen correlation coefficient strategy for correlation analysis amongst the chosen variables. The obtained Pearson plots for different variables during the aforesaid phases of the chosen period have been shown in Fig. 7.

During the pre-lockdown phase, the mortalities due to COVID-19 show a slight negative correlation with RH and PMs. The Spearmen correlation coefficient for COVID-19 mortalities was estimated to be harmful as RH (− 0.11), PM2.5 (− 0.23), and PM10 (− 0.34). Nevertheless, the correlation is small but cannot be stated as random as the estimated p-values for these correlations were less than 0.001. However, the observed correlation between COVID-19 mortalities with NH_3_ is insignificant, as the p-value for this correlation is 0.274. It anticipates a probability of a correlation between COVID-19 impact on RH and PM concentration in Delhi's atmosphere. The slight correlation values can be attributed to various factors contributing to COVID-19 impact, including free mobility of infected individuals and industrial and commercial activities contributing to more significant respiratory failures. Moreover, ammonia is the observed common factor linking coronavirus hotspots worldwide like bat caves, contaminated air in the proximity of slaughterhouses and agricultural fields treated with livestock farm sewage, and polluted air in megacities with air pollution. The direct role of ammonia is hypothesized to alkalinize the environment, which is favourable to coronavirus transmission^54^. It has been argued that the SARS-CoV-2 S protein undergoes conformational changes in the alkaline pH environment. Such an environment is required to induce the fusion of the virus with the plasma membrane of target cells^34^.

However, the number of COVID-19 cases shows a high negative correlation with PM_2.5_ (correlation = − 0.63 with p-value < 0.001) and RH (correlation = − 0.58 with p-value < 0.001) during the pre-lockdown phase. Moreover, the COVID-19 cases show a moderate negative correlation with PM_10_ (− 0.41, p-value < 0.001) and NH_3_ (− 0.33, p-value < 0.001). It anticipates a strong dependence of COVID-19 transmission majorly on PM and RH. The role of RH on COVID-19 transmission has already been discussed in the previous section, which validates our initial hypothesis of choosing RH as a significant variable in understanding its COVID-19 impact^55,56,64,72^. It has already been reported that secondary pollutants such as ammonia plays a significant role in COVID-19 transmission. It alkalizes the atmosphere, which is favorable for SARS-CoV-2 transmission and fusion of the virus with the plasma membrane of target cells^54^. Hence, there is a moderate correlation between a concentration of pollutants and RH with COVID-19 impact in the pre-lockdown Phase during the second impact wave in Delhi.

During the lockdown period, a significantly high positive correlation of COVID-19 caused deaths with PM_2.5_ (correlation = 0.60 with p-value < 0.001) and PM_10_ (correlation = 0.56 with p-value < 0.001). However, RH and ammonia show a high negative correlation with deaths with a correlation value of − 0.59 (p-value < 0.001) and − 0.35 (p-value = 0.023), respectively. The negative COVID-19 impact on RH again strengthens the hypothesis that low RH supports higher transmission and penetration of viruses and weakens human immunity^56,64^. However, the virus droplets bound to PM_2.5_ are more probable for deeper penetration in susceptible individuals' alveolar range, causing higher respiratory failures^27,36^. Hence, the obtained strong correlation amongst PM and COVID-19 mortalities is justified.

The number of COVID-19 cases during lockdown is also positively correlated with PM_2.5_ with a correlation value of 0.56 (p-value < 0.001) and PM_10_ with a correlation value of 0.51 (p-value < 0.001). It is attributed to the role of PM as an aerosol-based second route of SARS-CoV-2 transmission. The virus binds to PM particles and penetrates quickly, making an individual more susceptible to COVID-19 by inhaling infected PM particles^36^. However, the number of COVID-19 cases shows significantly high anti-correlation with RH (correlation = − 0.55 with p-value < 0.001). It again validates that lower RH supports further virus transmission. Hence, during the lockdown period, a high correlation exists between PM and RH with COVID-19 cases and mortalities.

During unlock period, no significant correlation was observed amongst COVID-19 caused mortalities and PM, RH and NH_3_. The estimated correlation value of COVID-19 caused deaths with PM_2.5_, PM_10_, RH and NH_3_ are 0.13, 0.04, − 0.10, and 0.23, respectively. However, their more significant p-values (0.32, 0.80, 0.52, and 0.12) predict a random correlation among them, which is not significant. It can be attributed to various factors contributing to COVID-19 mortalities apart from the chosen variables. There are also reports of delays in updating data related to COVID-19 mortalities, which has caused this trivial correlation. However, a weak positive correlation between COVID-19 cases with PM sustains in unlock period. The estimated correlation values of COVID-19 cases with PM_2.5_ is 0.33 (p-value of 0.029), and with PM_10_ is 0.34 (p-value of 0.024). It anticipates the persistence of dependence of COVID-19 transmission on PM’s concentration in Delhi’s environment. However, the correlation of COVID-19 cases with other variables is insignificant due to p value > 0.10.

**Figure 8:**
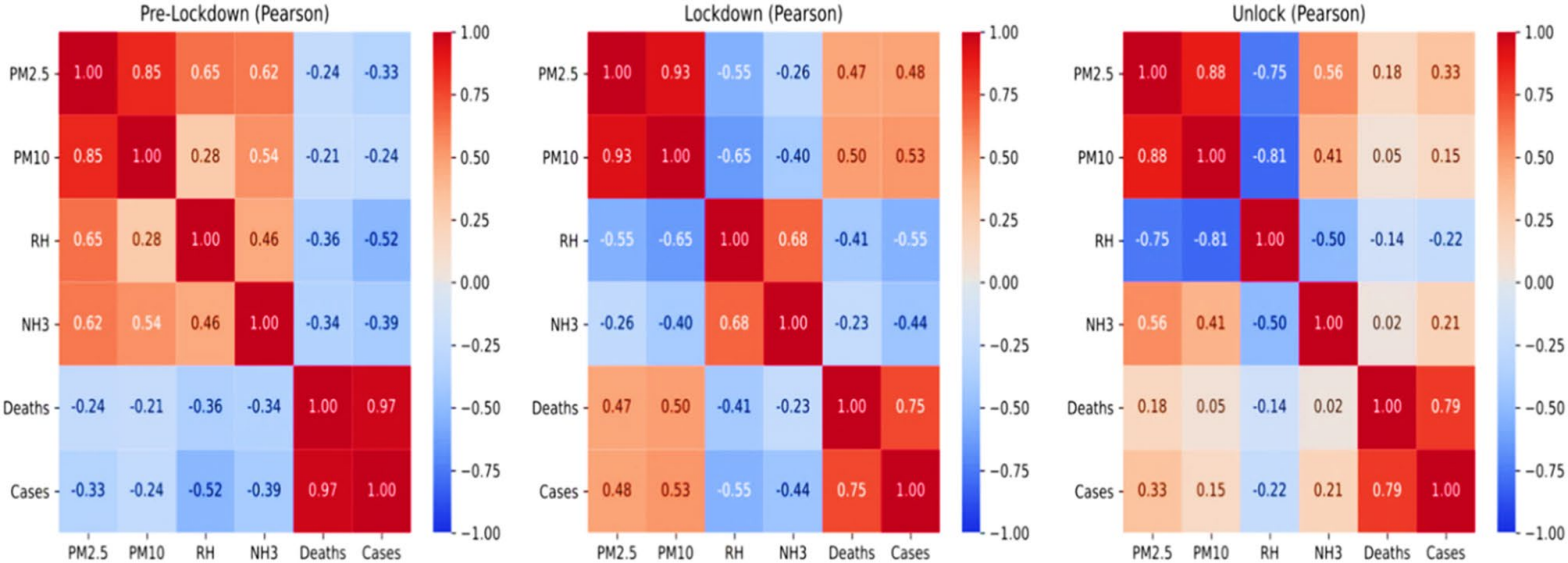
Pearson correlation plots amongst different variables, including environmental risk factors and COVID-19 severity for pre-lockdown phase, lockdown phase, and unblock phase.

Moreover, Pearson coefficient correlation has also been performed to evaluate correlation amongst the variables for comparative analysis with Spearmen strategy-based results^84–86^. The obtained Pearson correlation plots in the aforesaid period and phases have been shown in Fig. 8.

The obtained results from Pearson's coefficient correlation analysis are the following that obtained from Spearman's analysis. Moreover, the correlation coefficients between COVID-19 impact and other variables (RH, PMs, and NH_3_) obtained from Spearman are more significant than Pearson's during the lockdown period. It shows that the COVID-19 impact with pollutants (PMs and NH_3_) and RH are monotonic and not linear. It further strengthens the choice of Spearmen analysis over Pearson analysis for current studies and validates the estimated correlation results.

## Discussions and conclusions

**Figure 9:**
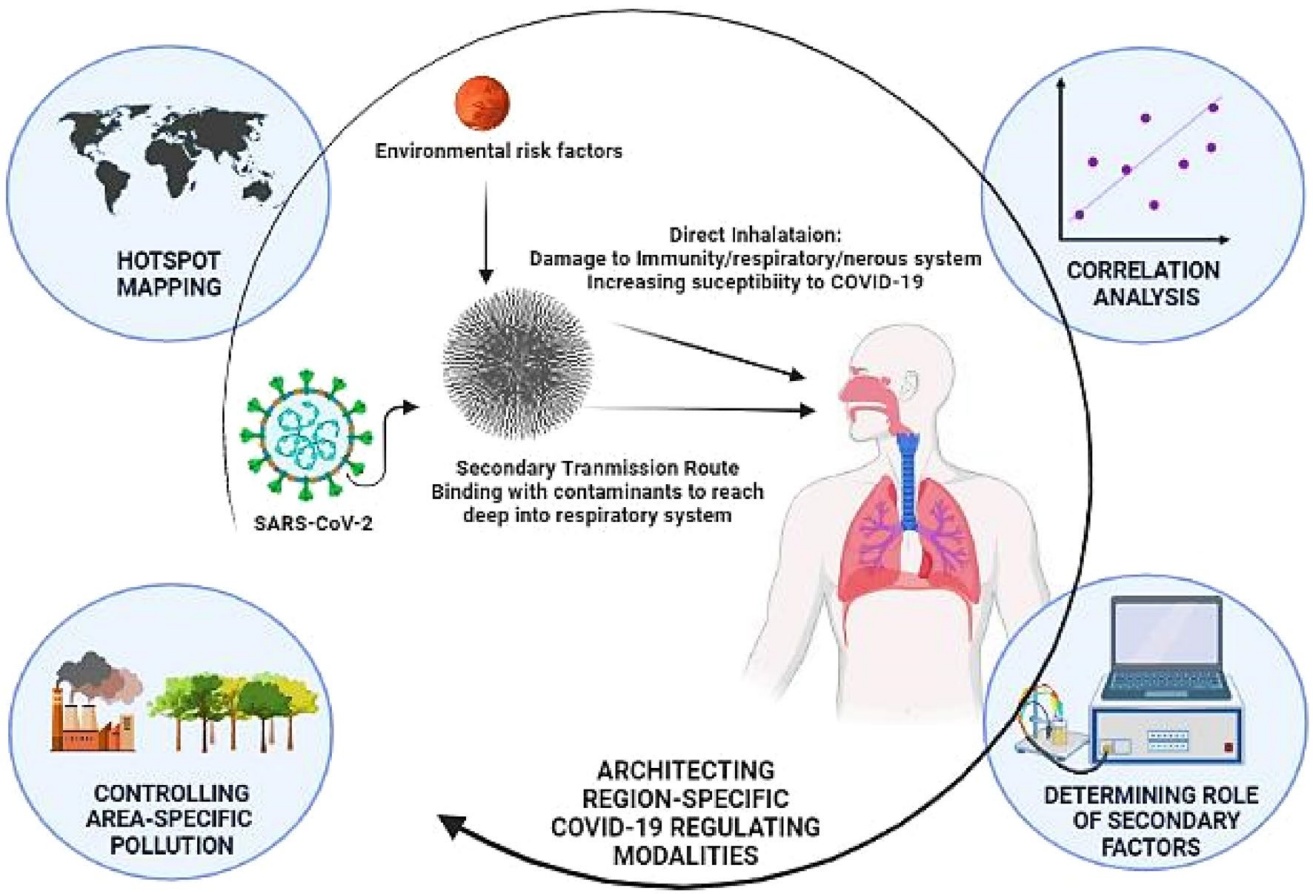
Schematic of outcomes of determining correlation between environmental risk factors and COVID-19 spread for designing area-specific regulating modalities for effectively combat the pandemic [created with BioRender.com].

In the present study, the impact of various environmental factors, including particulate matter, ammonia and relative humidity, on COVID-19 spread and mortality has been evaluated using various correlation analyses. It is evident that these factors vary regionally and play a vital role in COVID-19 progression and severity, which requires immediate attention. Depending upon the various outcomes of analyses, a scheme of conclusive outcomes to design area-specific COVID-19 regulations highlighted in Fig. 9 and is as following:

1. **Persistence of strong correlation between environmental variables and COVID-19 contagion:** The role of leading environmental risk factors (PM, RH, and NH_3_) for fatalities, coupled with their emergent evidence of association with COVID-19 contagion, motivates attention to assessing their concentrations during the second impact wave in Delhi. Assessments of ambient concentrations during the pandemic are required better to understand the relationship between COVID-19 and variable concentrations. However, gaps in ground-based monitoring, coupled with latency in available data, monotonic dependence of aforesaid variables on COVID-19 impact, motivate alternative Spearmen correlation coefficient strategy. Statistical correlation analysis indicated a correlation between COVID-19 impact (cases and mortalities) and environmental factors (PM, NH_3_, and RH) during the second impact wave in Delhi. The dependence of COVID-19 severity on RH and NH_3_ is found to be moderate (>0.4) and significant (p-value < 0.001) during the lockdown phase (19th April 2021 till 31st May 2021) in Delhi. It strongly predicts the potential role of RH and NH_3_ in SARS-CoV-2 outbreak through abating the immunity, stimulating receptor cell binding, and formation of aerosols for secondary transmission. The association of COVID-19 cases and mortalities with PM_2.5_ (r = 0.63 and 0.60) and PM_10_ (r = 0.41 and 0.56) is high and significant, anticipating PM as a significant risk factor promoting COVID-19 outbreak and severity. The associations detected in regression analyses provide strong justification for air contamination assisted severe COVID-19 outbreak in Delhi during the second impact wave.
2. **Hotspot mapping:** Moreover, the hotspots of airborne PM_2.5_, PM_10_, NH_3_, and RH in Delhi have been identified by mapping their ambient concentration. The reasoning for hotspots inferred from the regression model is based on identifying industrial and agricultural areas in Delhi. Primary PM hotspots are areas with a massive construction, vehicular emission, and industrial activities. Hotspots for secondary environmental factors (NH_3_ and RH) release are agricultural and green areas.
3. **Success of strategic lockdown:** Estimations from the regression model during strategic lockdown have clearly suggested the notable success of unprecedented restrictions in Delhi in the form of economic shutdowns and limitations on mobility. The estimated decrease in ambient contaminant concentration observed in regression analyses provides strong justification for the lockdown success rate in Delhi. Nevertheless, the resulting economic crisis and degrading mental health of confined people are the foremost associated concerns.
4. **Prospects:** Estimated outcomes from this study, including correlation amongst variables, hotspot identification, and lockdown success, must be interrelated to design COVID-19 monitoring and regulating policy effectively. The assessed temporal and spatial variation in COVID-19 impact based on variation in ambient concentration of environmental risk factors has strongly suggested the requirement of area-specific regulatory modalities. The first step is to map the hotspot of COVID-19 severity and environmental risk factors and estimate their interrelation. The regulatory measures to effectively control the contributing environmental factors must be employed in furtherance to decrease COVID-19 severity and associated fatalities. The outcomes from the study also strongly anticipate the requirement of an acceptable ammonia monitoring policy for agricultural practices, monitoring of indoor RH to slow down viral contagion, and compelling urban and transportation planning to regulate PM emissions.

Delhi being one of the top polluted cities in the world has faced a severe COVID-19 outbreak during the second wave of the pandemic. In spite of strict lockdown, continuous monitoring and large-scale vaccination, the severity caused during the second wave was devastating. It strongly suggests the requirement to improve present regulating policies such as vaccination drive and forming area-specific new policies to control the infection effectively. This study has revealed the area-specific environmental factors contributing to the severity of COVID-19. The obtained outcomes strongly anticipate that there exists a strong correlation between environmental factors, especially PM and COVID-19 spread and severity. All the studied environmental factors play a specific role in amplifying COVID-19 severity either by turning humans susceptible to the infection or acting as secondary carriers promoting its spread. Moreover, secondary contaminants (RH and NH_3_) play a significant role in affecting the immunity, promoting receptor cell binding, and creating a secondary transmission route by promoting the formation of aerosols. However, there is notable variation in regional concentration of these environmental factors, which suggests designing the area-specific COVID-19 monitoring and control strategies. It includes  mapping pollution and COVID-19 hotspots, analyzing correlation amongst the two variables, implying strategic lockdown in hotspots, adopting area-specific measures to control the concentration of supportive environmental factors towards COVID-19 pandemic, and strategic area-specific vaccination. Hence, a collective approach from policy-makers, health workers, data scientists, and environmentalists is required to design and implement region-specific policies to control future COVID-19 waves and public health emergencies due to virus outbreaks and similar infectious diseases.

## Materials and methods

### Study area

In the current study, 'Delhi’, the capital city of India, also one of the highly populated and polluted metropolitan capitals, has been selected for analysis. Delhi is located at 28.61° N 77.23° E and holds the second position in the world's leading megacities^87^. It is India’s most significant urban city with more than 15 million population and a population density of around 11,297 people per km^2^^88^. The burning of biomass (field straws) and vehicular emissions are the primary sources of PM in ambient air in Delhi^41^. However, the agricultural sector majorly contributes to airborne ammonia^79,80^. The total number of registered vehicles was the highest (10.26 million in 2017) compared to other Indian metropolitan cities^89^.

### Data collection and processing

The data relating to the concentration of various air contaminants particulate matter PM2.5, PM10, ammonia (NH_3_), and relative humidity (RH) has been collected from 17 ground monitoring stations across various sub-districts in Delhi (https://app.cpcbccr.com/ccr/#/caaqm-dashboard-all/caaqm-landing). It examines the environmental impact of the COVID-19 cases, deaths, and the government's prevention measures before and after the second wave in 2021. The data was collected for a period ranging from 1st January 2021 to 14th July 2021 from the Central Pollution Control Board (CPCB), under the Ministry of Environment, Forests and Climate Change, Government of India (https://cpcb.nic.in/). The daily covid cases and deaths of the aforementioned period were procured from the WHO (https://covid19.who.int/). The daily COVID deaths, cases, and pollutants, particulate matter PM2.5, PM10, NH_3_, and RH, were analyzed from 1st January 2021 to 14th July 2021.

### Missing data processing

The prevalent issue in air quality analysis is the incapability of various statistical analysis strategies to cater to missing observations^90,91^. The sources for missing in-situ air pollutant data observations include power outages, filter changes, malfunctions and errors, pollutant concentrations lower than detection limits, and computer system crashes^91^. The problem of missing data is pervasive and can affect the analysis significantly if not appropriately treated. Previous reports on air quality data analysis have identified three different classes of missing observation scenarios, including missing at random (MAR), missing completely at random (MCAR), and not missing at random (NMAR)^91,92^. The data set used in the present studies was the MAR criterion for 7% of missing data.

The most commonly used method for handling missing data is the "last observation carried forward” (LOCF) method. In the LOCF method, the missing value in the data is replaced with the previous observation of the same variable^93^. The method assumes that the variable's value remains unchanged for the missing data, and missing data is replaced with the last observed value.

The analysis has been performed in two parts. The first part is the statistical and comparative analysis of the air quality data of each 17 locations within Delhi from January to July 2021. This part discusses the air quality index (AQI) across different sub-districts of Delhi and its temporal variation.

The second part is focused on the effect of the COVID-19 spread, mortalities, restrictions, and lockdowns on the AQI of Delhi. The AQI of Delhi is taken as the average of the AQI over the 17 locations. The period from January to July 2021 is divided into three stages; the first stage, from 1st January 2021 to 18th April 2021, is the pre-lockdown period. Due to the high number of COVID-19 cases and mortalities, the Delhi government imposed a complete lockdown from 19th April 2021 till 31st May 2021, which comprises the second lockdown stage. The third stage is the unlock period from 1st June 2021 till 14th July 2021. The unlock was done in 5 stages in a piece-wise manner till the complete services were restored. The entire unlock period has been considered as a single stage in the current study. The above regression methodology has been employed to fill in the missing data observations for AQI datasets and COVID-19 data sets utilized in the current study.

### Methodology for data modeling: Spearmen and Pearson correlation coefficient

Spearman's rank correlation coefficient analysis, a non-parametric measure of the rank correlation between two variables by assessing the monotonic relationship between the variables, has been used in the present analysis^86^. However, previous reports on similar correlation analysis have used the Pearson correlation coefficient test. The main difference between Spearman's rank correlation and the most commonly used Pearson correlation coefficient is that the Pearson correlation coefficient can evaluate only the linear relationship between the variables^85^. In contrast, Spearman's rank correlation benchmarks a monotonic relationship. If the Spearman correlation coefficient is greater than the Pearson correlation coefficient, the correlation between the variables is monotonic but not linear. Since the collected data for COVID-19 and AQI for Delhi in the present analysis is not normally distributed, Spearman's rank correlation coefficient is anticipated to give relevant results with high significance.

The Spearman correlation coefficient is defined as the correlation between the rank variables^86^. Consider two variables (X_i_, Y_i_) which are first converted to rank variables (R(X_i_), R(Y_i_)), the Spearman rank coefficient (ρ_s_) is defined as

$${\rho }_{s}=\frac{cov(R\left(X\right),R\left(Y\right))}{[{\sigma }_{R\left(x\right)}{\sigma }_{R\left(Y\right)}]}$$

where Cov(R(X), R(Y)) is the covariance of the rank variables. σ_R(X)_ and σ_R(Y)_ are the standard deviations of the rank variables.

If in the sample of size ‘n’, all n ranks are distinct, then the Spearman rank coefficient (ρ_s_) is given by

$${\rho }_{s}=1-\frac{6\sum {d}_{i}^{2}}{n({n}^{2}-1)}$$

where d_i_ = R(X_i_) – R(Y_i_) is the difference between the rank of each observation between two variables. The correlation coefficients lie in the typical range − 1 (positive correlation) to + 1 (anti-correlation).

### Significance test

The probability value (p-value) has been calculated to analyze the significance of statistical correlation amongst the mentioned variables^94,95^. The p-value measures the extent of random correlation between the given variables and ranges between 0 to 1. For instance, the p-value of 1 implies a 100% probability of random correlation amongst the given variables. It is stated as a 'null hypothesis' illustrating no existing significant correlation between the given data sets other than randomness. A p-value close to zero signifies that the null hypothesis is false and a significant pure correlation amongst the given variables. Hence, as the p-value approaches zero, the significance of obtained correlation amongst the variables is purer and not random.

## Data Availability

The respective data used in the present analysis are available on CPCB, DPCC, and WHO websites.
